# Angiomatous Meningioma in the Craniocervical Junction

**DOI:** 10.1155/2021/6651160

**Published:** 2021-04-10

**Authors:** Shunsuke Ito, Yoichi Iizuka, Masanori Aihara, Hiromi Koshi, Tokue Mieda, Eiji Takasawa, Sho Ishiwata, Yusuke Tomomatsu, Akira Honda, Kazuhiro Inomata, Hirotaka Chikuda

**Affiliations:** ^1^Department of Orthopaedic Surgery, Gunma University Graduate School of Medicine, 3-39-22, Showa, Maebashi, Gunma 371-8511, Japan; ^2^Department of Neurosurgery, Gunma University Graduate School of Medicine, 3-39-22, Showa, Maebashi, Gunma 371-8511, Japan; ^3^Clinical Department of Pathology, Gunma University Hospital, 3-39-22, Showa, Maebashi, Gunma 371-8511, Japan

## Abstract

**Introduction:**

Spinal angiomatous meningioma arising in the craniocervical junction has not been reported. *Case Presentation*. A 68-year-old man presented to our hospital with pain in the back and left leg. He showed slight motor weakness in his upper extremities. Magnetic resonance imaging revealed a mass with marked enhancement in the craniocervical junction. Computed tomography angiography showed feeding vessels arising from the right vertebral artery. Preoperative embolization of the feeding vessels was performed to reduce intraoperative bleeding. Gross total resection of the tumor was achieved by debulking and piecemeal resection. The tumor attachment to the dura mater was also resected (Simpson grade 1 resection). A histopathological examination confirmed the diagnosis of an angiomatous meningioma. The patient's symptoms improved shortly after surgery.

**Conclusions:**

We achieved gross total resection of spinal angiomatous meningioma arising in the craniocervical junction. A preoperative evaluation and embolization of the feeding arteries may help prevent massive intraoperative bleeding.

## 1. Introduction

Angiomatous meningioma, characterized by an abundant vascular component, is a rare subtype of meningioma, accounting for 2.1% of meningiomas [1]. Angiomatous meningiomas arising from the spinal cord is even rarer. We herein report a case of angiomatous meningioma in the craniocervical junction.

## 2. Case Presentation

**Figure 1:**
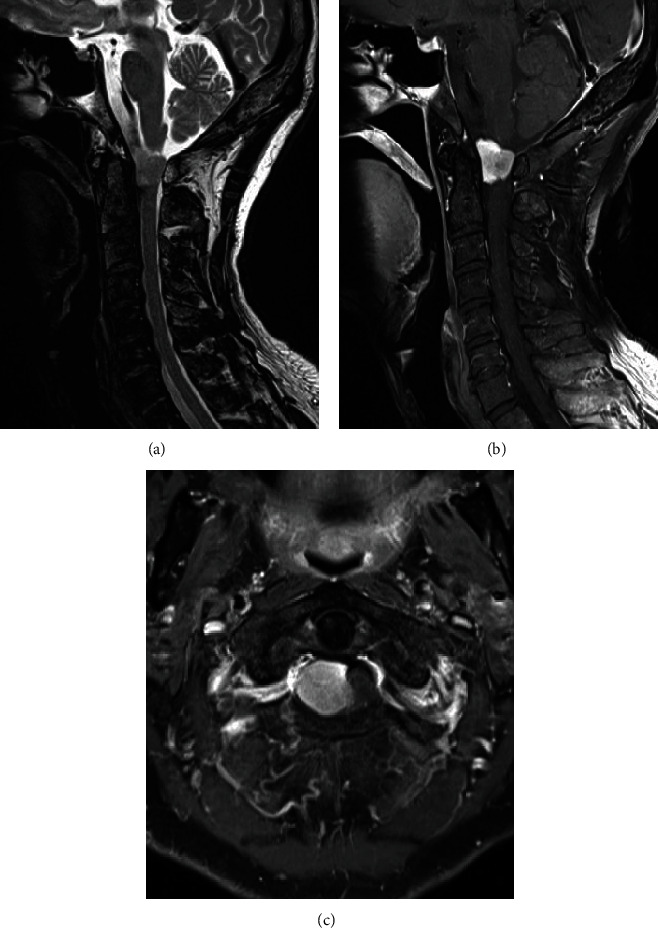
(a) T2-weighted sagittal MRI of the cervical spine showed a tumor in the craniocervical junction. (b, c) MRI followed by the intravenous administration of gadolinium-diethylenetriaminepentaacetic acid (Gd-DTPA) showed a homogeneously enhanced intradural tumor without obvious dural tail sign.

A 68-year-old man without notable comorbidity presented to our hospital with a 15-month history of pain in the back and left leg. A neurological examination revealed motor weakness (manual muscle testing 4 out of 5) in the upper extremities. Magnetic resonance imaging (MRI) demonstrated an intradural mass lesion at the craniocervical junction, which has low intensity on both T1- and T2-weighted imaging. Enhanced MRI with gadolinium-diethylenetriaminepentaacetic acid (Gd-DTPA) showed homogeneous enhancement without a dural tail sign (Figure 1). Computed tomography angiography using iodinated contrast agent revealed two feeding vessels arising from the right vertebral artery.

**Figure 2:**
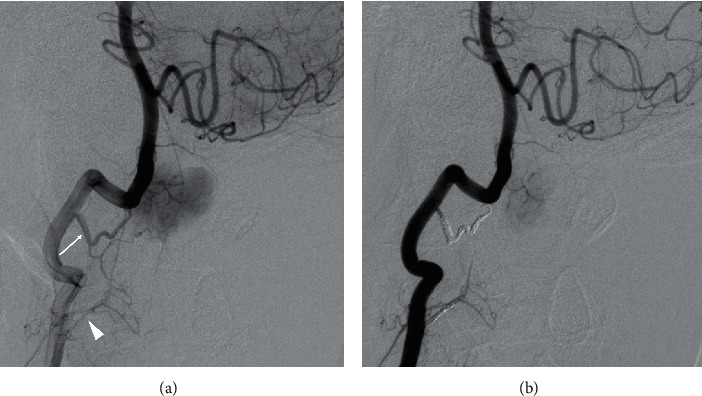
(a) Right vertebral angiogram demonstrating tumor staining, supplied by the right C2 (arrow) and C3 (arrowhead) segmental arteries. (b) A right vertebral angiogram after embolization using a coil showing slight tumor staining.

**Figure 3:**
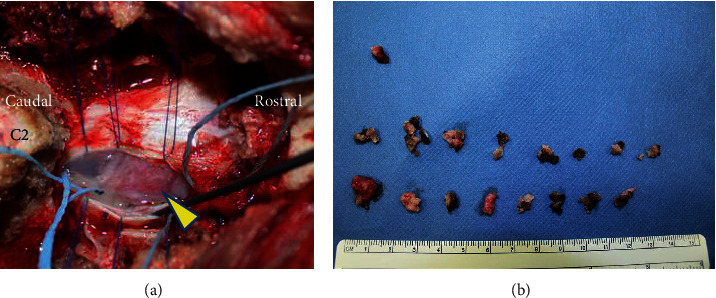
(a) A mass was identified after durotomy (arrowhead). (b) The tumor was resected in a piecemeal fashion.

Given the aggravating symptoms, the patient decided to undergo surgery. To reduce the risk of massive bleeding, we embolized the feeding arteries using soft coils prior to surgery. An angiogram after embolization showed a decreased tumor stain (Figure 2). Subsequently, resection of the tumor was performed the same day. Following durotomy, a tumor mass was identified on the ventrolateral side of the spinal cord. Resection was carefully performed via central debulking with piecemeal resection to avoid neurological damage under spinal monitoring (Figure 3). We observed substantial but manageable bleeding from inside of the tumor. The tumor attachment to the dura mater was also resected (Simpson grade 1 resection). The dural defect was repaired with a Gore-Tex patch and further covered with a bioabsorbable polyglycolic acid sheet and fibrin glue. The intraoperative blood loss, mainly due to bleeding from the tumor itself, was 567 ml. The drain was removed two days after surgery without noticeable cerebrospinal fluid leakage.

**Figure 4:**
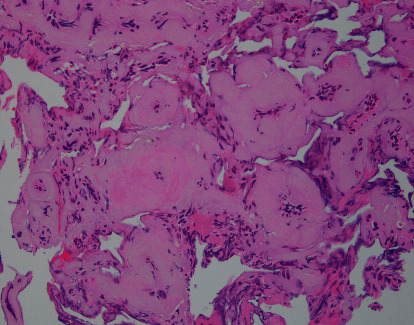
The resected tumor was composed of numerous blood vessels and intervening tumor cells. The blood vessels were variably hyalinized and thick-walled.

A histopathological examination showed numerous blood vessels making up most of the mass and intervening tumor cells. The blood vessels were variably hyalinized and thick-walled. The tumor cells had round nuclei with occasional intranuclear pseudoinclusions. Some whorls and psammoma bodies were present (Figure 4). Immunohistochemically, the tumor cells were positive for epithelial membrane antigen, and the MIB-1 index was <1%. The tumor was diagnosed as an angiomatous meningioma.

**Figure 5:**
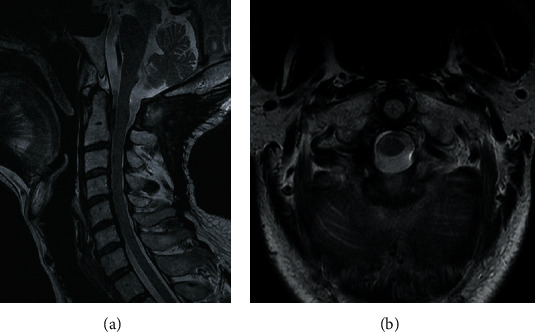
(a, b) T2-weighted sagittal and axial MRI at one year after surgery showed no obvious recurrence.

The patient's symptoms, including back pain and leg pain, improved shortly after surgery. MRI obtained one year after surgery revealed decompression of the spinal cord and no sign of recurrence (Figure 5). The patient remained neurologically intact until he died of an unrelated cause 16 months after surgery.

## 3. Discussion

Spinal angiomatous meningioma is extremely rare. To our knowledge, a total of 16 cases have been reported (Table 1) [2–6]. Angiomatous meningiomas are classified as WHO grade 1 meningioma without aggressive nature, while motor and/or sensory deficits and bladder and rectal disturbance can occur as the disease progresses. Spinal angiomatous meningiomas arise most commonly in the thoracic spine followed by the cervical spine [2–6]. The present case is the first report of angiomatous meningioma in the craniocervical junction.

The preferable treatment for angiomatous meningioma is Simpson grade 1 resection (total resection including dural attachment and abnormal bone) although some researchers suggested that Simpson grade 2 resection (total resection and dural coagulation) is sufficient [4, 7–10]. In the present case, we decided to perform Simpson grade 1 resection to reduce risk of potential recurrence. As angiomatous meningiomas are rich in small blood vessels, preoperative embolization to reduce tumor blood supply may be useful [11]. Contrarily, Wu et al. reported good control of intraoperative bleeding without using preoperative embolization. The authors found that en bloc resection was associated with a significant reduction of intraoperative blood loss compared with piece-by-piece resection [4]. In the present case, even though we successfully embolized the feeding arteritis just prior to the surgery, we still observed substantial intraregional bleeding.

When spinal angiomatous meningioma is suspected, we recommend performing a preoperative evaluation of the feeder vessels using an angiogram. Subsequent embolization of the feeder vessels should be discussed, especially when the tumor is located ventrally, making en bloc resection difficult.

## Figures and Tables

**Table 1:** Summary of 17 cases of angiomatous meningioma of the spinal cord and the present case.

Authors	Age	Sex	Location	Treatment	Dural attachment	Blood loss	Follow-up	Outcome
Gul et al. 2008 [2]	25	F	T9-L2	GTR	NA	NA	NA	NA
Vij et al. 2012 [3]	16	M	C2-C5	GTR; piecemeal resection	NA	NA	3 d	Dead
Wu et al. 2015 [4]	28	M	C2-C3	Simpson grade II; piecemeal resection	Ventral	400	152 m	No recurrence
	56	F	C1-C3	GTR; en bloc resection	Nerve root	100	131 m	No recurrence
	54	F	T10-T11	Simpson grade II; piecemeal resection	Ventral	500	120 m	No recurrence
	49	M	T1-T3	Simpson grade I; en bloc resection	Dorsal	200	108 m	No recurrence
	43	F	T1	GTR; en bloc resection	Nerve root	100	99 m	No recurrence
	28	F	C2-C4	GTR; en bloc resection	Nerve root	100	86 m	No recurrence
	67	F	C7-T3	Simpson grade II; en bloc resection	Lateral	300	72 m	No recurrence
	76	F	T4	Simpson grade II; en bloc resection	Lateral	100	61 m	No recurrence
	41	M	T11-T12	Simpson grade II; en bloc resection	Lateral	100	53 m	No recurrence
	54	M	C1-C2	Simpson grade II; piecemeal resection	Ventral	600	32 m	No recurrence
	57	M	T12-L1	Simpson grade II; piecemeal resection	Lateral	400	18 m	No recurrence
	42	M	C2-C3	Simpson grade II; en bloc resection	Lateral	200	11 m	No recurrence
Yang et al. 2016 [5]	55	F	T6-T8	Simpson grade II; en bloc resection	Dorsolateral	NA	6 m	No recurrence
Missori et al. 2017 [6]	40	M	T3-T4	GTR	Nerve root	NA	14 m	No recurrence
Present case	68	M	O-C1	Simpson grade I; piecemeal resection	Ventrolateral	567	11 m	No recurrence

M: male; F: female; C: cervical; T: thoracic; L: lumbar; GTR: gross total resection; RT: radiotherapy; CT: chemotherapy; NA: not available; d: day; m: month.

## Data Availability

There are no available data supporting the results of this study as it is a case report.
